# Virtuous organizations: Desire, consumption and human flourishing in an era of climate change

**DOI:** 10.3389/fsoc.2022.960054

**Published:** 2022-11-24

**Authors:** Geoff Moore

**Affiliations:** Department of Management and Marketing, Durham University Business School, Durham University, Durham, United Kingdom

**Keywords:** Anthropocene, climate change, consumption, desire, Girard, MacIntyre, virtuous organizations

## Abstract

The notion of virtuous organizations has an established place in the business ethics/organization studies literature. But this conceptualization drew principally on Alasdair MacIntyre's *After Virtue*. His more recent work *Ethics in the Conflicts of Modernity*, with its focus on desire, consumption and human flourishing, demands a revisiting of the original concept. The first aim of this paper, therefore, is to provide an extended theory of the notion of the virtuous organization. An obvious application of this extended theory is to the issue of climate change. In exploring this, the paper has a further aim which is to respond to Banerjee et al.'s call for more theory building that articulates post-growth possibilities at the organization level in relation to the multiple challenges which society faces in response to the changing climate. The paper begins by summarizing the current conceptual framework of the virtuous organization while recognizing critiques of MacIntyre's work and its organizational application. It then turns to the issues of desire and consumption highlighted in MacIntyre's latest book, drawing also on an extended literature in these areas including insights from Girard's work, and concluding with MacIntyre's contentions in relation to human flourishing. This leads to the extended conceptual framework which is then applied to the issue of climate change. The particular theoretical contribution of the paper is to understand virtuous organizations as playing an important role in the redirection and re-education of desires, leading to the pursuit of goods that we have good reason to desire, and so to *the* good for individuals and communities, and ultimately to human flourishing within ecological limits. The similarities with and differences from the degrowth/post-growth movement are explored to demonstrate the distinctive contribution a MacIntyrean approach makes. The practical implications of this theoretical contribution are then spelled out, including a consideration of the potential ubiquity or otherwise of this approach, before conclusions are drawn.

## Introduction

The conceptual construct of the virtuous organization, based on the work of the moral philosopher Alasdair MacIntyre (1981/2007), has an established place in the business ethics/organization studies literature. This literature contains both theoretical and empirical contributions focused mainly on business organizations (see, for example, Moore, 2005, 2012a, 2017; Moore and Beadle, 2006; von Krogh et al., 2012; Bernacchio, 2018; Sinnicks, 2019; Chu and Moore, 2020; Rocchi et al., 2021). It has, however, not yet incorporated insights from MacIntyre's (2016) latest book *Ethics in the Conflicts of Modernity*, insights that lead to a significant extension of the construct centring on the nature of desire, its fulfillment in consumption, and its relationship to human flourishing, and the organizational implications of this. The first aim of this paper, therefore, is to build an extended theory of the virtuous organization.

The paper then addresses how this extended theory might be applied to the issue of climate change. In exploring this, the paper has a further aim which is to respond to Banerjee et al.'s call, in relation to the multiple contemporary challenges which society faces, for “more theory building that articulates post-growth possibilities at the organization level” (Banerjee et al., 2021, p. 351). The particular theoretical contribution of this paper is to understand organizations as playing an important role in the redirection and re-education of desires, leading to the pursuit of goods that we have good reason to desire, and so to *the* good for individuals and communities, and ultimately to human flourishing within ecological limits.

The paper begins by summarizing the current conceptual framework of the virtuous organization while recognizing critiques of MacIntyre's work and its organizational application. It then turns to the issues of desire and consumption highlighted in MacIntyre's latest book, locating this within an extended literature in these areas, and concluding with MacIntyre's contentions in relation to human flourishing. This leads to the extended conceptual framework which is then applied to the issue of climate change.

The similarities with and differences from the degrowth/post-growth movement (Banerjee et al., 2021) are explored to demonstrate the distinctive contribution a MacIntyrean approach makes. The practical implications of this theoretical contribution in relation to climate change are then spelled out, including a consideration of the potential ubiquity or otherwise of this approach, before conclusions are drawn.

## Virtuous organizations—The story so far

Alasdair MacIntyre's importance as a philosopher of virtue is undisputed, and he is the most widely cited writer after Aristotle in the field of virtue ethics in business (Ferrero and Sison, 2014). He is extensively read outside philosophy as a recent volume *Learning from MacIntyre* (Beadle and Moore, 2020), with chapters ranging from theology through sociology and education to therapeutic methods, illustrates. Unsurprisingly, his work is not without challenge, Solomon (2003) arguing that these fell into two broad camps: those who claimed that MacIntyre was too pessimistic in his analysis of contemporary society; and those who contended with his alternative, Neo-Aristotelian, and more recently Thomistic, proposals. For our purposes, while there were early criticisms of his characterization of the Manager in *After Virtue* (MacIntyre, 1981/2007) (for example, Mangham, 1995), and of his critique of bureaucratic organizations (for example, Du Gay, 1998), criticisms of the proposals for the organizational application of his work which concerns us here are few and largely internal to the debate (Sinnicks, 2019), or are extensions of it to broader societal concerns (Bernacchio, 2018). We turn, therefore, to the current conceptual framework without need for modification.

It has been argued that organizations of all types, including business organizations, can be characterized, using MacIntyre's (1981/2007) terminology, as practice-institution combinations (Moore and Beadle, 2006; Moore, 2012a,b, 2017). A practice is:

“any coherent and complex form of socially established co-operative human activity through which goods internal to that form of activity are realized in the course of trying to achieve those standards of excellence which are appropriate to and partially definitive of, that form of activity...” (MacIntyre, 1981/2007, p. 187)^1^

This is not to say that all such organizations necessarily house practices (see Moore, 2017, p. 142–146), but where they do they will have in good order the pursuit of three different kinds of goods. The first such kind, which forms part of the definition above, is internal goods which comprise both the excellence of the products or services that the organization provides, and the “perfection” of the individual practitioners in the process (MacIntyre, 1994, p. 284; see also MacIntyre, 1981/2007, p. 189–190—while MacIntyre does not qualify or define “perfection,” it might be taken to accord with the notion of human flourishing which is discussed below).

However, while the pursuit and achievement of such internal goods might seem to be a good in itself (subject to the condition that individual practitioners will need to identify which practices lead to *their* flourishing under the particular circumstances of *their* lives), there is a further qualification which needs to be made. This is the extent to which the internal goods of the particular practice in question contribute to the good of the community: “The common goods of those at work together are achieved in producing goods and services that contribute to the life of the community” (MacIntyre, 2016, p. 170). And the determination and achievement of such common goods will be one function of the virtuous organization, where practitioners deliberate “with others as to how in this particular set of circumstances here and now to act so as to achieve the common good of this particular enterprise” (MacIntyre, 2016, p. 174). This will, however, also require wider deliberation beyond the bounds of the organization itself: “In contemporary societies our common goods can only be determined in concrete and particular terms through widespread, grassroots, shared, rational deliberation” (MacIntyre, 2010). And such deliberation will need to synthesize the inherent feature of practices to be creative—and thereby develop new products and services which serve society—with the need, anticipating the climate change application below, to contribute to ecological and eco-system sustainability in so doing.

In addition to the pursuit of internal and common goods, however, is a third kind of goods—external goods. These goods, goods such as survival, reputation, power, profit and, more generally, success, are the particular concern of the institutional element of the practice-institution combination. Institutions, on MacIntyre's terms, are:

“characteristically and necessarily concerned with... external goods. They are involved in acquiring money and other material goods; they are structured in terms of power and status, and they distribute money, power and status as rewards. Nor could they do otherwise if they are to sustain not only themselves but also the practices of which they are the bearers.” (MacIntyre, 1981/2007, p. 194)

External goods are required to enable the survival and development of the organization and the core practice at its heart—its flourishing. Indeed, external goods depend upon the realization of internal goods since they arise from the revenue derived from the goods or services produced by the practice. There is, thus, an “essential but complex circularity between internal goods and external goods” (Moore, 2012a, p. 380). But this is not to say that this complementarity implies equality. There is a hierarchy of goods, such that external goods, while necessary, are to be subordinated to both internal and common goods. And, as has been noted previously (Moore, 2012a), this imposes on organizations the requirement both to *order* the different kinds of goods appropriately, while achieving *balance* in their pursuit. It is, for example, possible for the pursuit of growth of the institution *for its own sake* (perhaps to achieve greater market share and hence power over its supply chain), to be such as to distort the sustenance and development of the practice—the Volkswagen “Dieselgate” scandal (Rhodes, 2016) perhaps being a case in point.

This theoretical conceptualization of organizations as practice-institution combinations, pursuing internal and common goods, while also requiring the balanced and ordered pursuit of external goods, a conceptualization which also identifies an inherent tension between the two ‘parts' of the organization, has led to the development of what has been termed the virtuous organization. And the core characteristics of such an organization, an organization that “crowds-in” virtue (Moore, 2012b) have been identified (see Moore, 2012a, p. 366, for example) as one which: has a *good purpose* realized in its pursuit of internal and common goods; recognizes that its most important function is *the sustaining and encouragement of excellence in the particular practice it houses*; and has an *ordered focus on the achievement of external goods*. Among the other characteristics of the virtuous organization (see Moore, 2005, 2017) is the development of power-balanced internal structures which require carefully designed systems of participation and self-governance (see Moore, 2012b, p. 310), and these will also be of importance when considering the practical implications of the extended theory below.

## Desire, consumption and human flourishing

As noted above, it is the consideration of desire and, to a lesser extent, consumption in MacIntyre's latest book that lies behind the need to reconsider and extend the current conceptual framework. But also as noted above, this needs to be located within the broader literature on desire, although a literature which is largely consistent with MacIntyre's contentions. We begin with a broad consideration of the nature of desire.

“Desire is a, if not the, basis of the human condition” (O'Shea, 2002, p. 938).^2^ While this may be so, it was Frankfurt's contention that desire is by no means confined to humanity, and that many other species similarly have what he termed “first-order desires” (Frankfurt, 1971, p. 7). What distinguishes humans, according to Frankfurt, is that they are able to form “second-order desires”—“no animal other than man [*sic*]... appears to have the capacity for reflective self-evaluation that is manifested in the formation of second-order desires” (Frankfurt, 1971, p. 7). Indeed further, what is essential for someone to be a person is that they have “second-order volitions” (Frankfurt, 1971, p. 10), in other words that they want a certain desire to be their will.^3^

Desire may be distinguished from needs and wants (Belk et al., 2003), although the distinctions are neither always clear-cut nor observed by all commentators. Thus, for example, McPherson refers to the “desire for nourishment, rest, affection, and security” (McPherson, 2019, p. 391) which might just as well be categorized under needs, since need “demonstrates an initial closedness... rooted in a *lack* of a certain category of objects” (Belk et al., 2003, p. 328, emphasis added). Want, however, “is normally taken as an expression of a personal, psychological preference structure” and is “too reassuringly controlled by the mind for it to cover the passionate aspects of desire” (Belk et al., 2003, p. 328). By contrast, then, desire is “deeply linked to the social world... [and] addresses the interplay of society and individual, of bodily passions and mental reflection” (Belk et al., 2003, p. 328–329). By way of definition, desire is *passionate imagining shaped by and expressed in a given social context* (see Belk et al., 2003, p. 329). McPherson makes a further useful distinction between desires that are innate (“‘hardwired' into our nature”), as opposed to those desires which are “an acquired disposition” (McPherson, 2019, p. 391), a distinction to which we will return.

Desires come in all shapes and sizes, including for a cup of coffee, to visit a friend before she dies, to own and continue owning a Bugatti, to see a food bank flourishing, to hope that something bad happens to a fraudulent salesman (see MacIntyre, 2016, p. 2). Desires, at least for adults, can also be future-oriented, so that actions now may determine the possibility of satisfying future desires in the medium- to long-term, and forgoing present desires may leave open future possibilities (see MacIntyre, 2016, p. 3).

However, as well as at least some desires being future-oriented, desires also have histories, a history “during which objects of desire have multiplied... some... earlier desires have been transformed, others replaced” (MacIntyre, 2016, p. 3). Thus a particular desire can be set aside or metamorphose into other desires, and such new desires can arise related to the development of our “emotions, tastes, affections, habits and beliefs” as well as “our biochemical and neurophysiological development” (MacIntyre, 2016, p. 3). Desire and the ability to reflect on, to seek to fulfill, to choose not to seek to fulfill, or not yet to seek to fulfill our desires, is therefore fundamental to the human condition. Yet, despite the nature and breadth of desires, desire and its fulfillment have become increasingly associated with consumption, and it to this that we now turn.

The fulfillment of desire is often, though not always, through consumption. We consume a cup of coffee, a holiday, a Bugatti (at least in the sense of possession and use). While we do not normally consider the satisfaction of a desire for a well-stocked food bank, for something bad to happen to a fraudulent salesman, for a romantic liaison, as things we consume, in some (perhaps slightly distorted) sense we could. But if desire is fundamental to the human condition, so too is consumption and the possessions (even temporary “possessions” such as food and drink) which go alongside it—“knowingly or unknowingly, intentionally or unintentionally, we regard our possessions as parts of ourselves,” they “contribute to sense of self” (Belk, 1988, p. 139, 160).

The focus, then, is on the consumption of “material” objects, though including the consumption of experiences, and on the explanation for why our desire for such goods seems to suffer from a “*ratchet effect*”; that there seems to be “an intrinsic human tendency toward the *escalation* of desire, through habituation to increasingly high-quality food, clothes and levels of comfort” (Michaelis, 2006, p. 329, emphasis added). And, as noted in relation to our sense of self, the reason seems to be related to

“the tendency to imbue material things with social and psychological meanings... Consumer goods provide a symbolic language in which we communicate continually with each other, not just about raw things, but about what really matters to us: family, friendship, sense of belonging, community, identity, social status, meaning and purpose in life.” (Jackson, 2017: 69)

Belk et al. (2003, p. 331) note that “social control of consumer desire, either in the form of external control or self-control, is thought to decline in a consumer society. Furthermore, a globalizing ethos of consumption promotes consumer desires and objects of desires.” Largely underlying such promotion of consumer desires and, as noted above, the ratchet effect, is Girard's notion of mimetic desire (see Belk et al., 2003, p. 329; Girard, 1966, 1977). As above with the difference between needs, wants and desire, Girard distinguishes between needs and appetites which are natural, on the one hand, and desires which are socially and culturally conditioned, on the other. Thus, our need for food, clothing, shelter and so on are not of immediate concern to his argument unless they become objects of competition. Girard's key contention in relation to our desires, however, is that, *contra* Freud (see Girard, 1977, p. 169–222), *our desires are not original to ourselves*, but are learned by imitation from others (and are thus an “acquired disposition”—see McPherson, 2019, above), such others thereby becoming both models (we come to desire what they desire), and potentially rivals (we cannot always attain it if the object is subject to competition). As Belk et al. note, commenting directly on Girard's work, “the basis for this competitive and emulative desire is a battle for prestige” (Belk et al., 2003, p. 329). Indeed, Girard claims that “desire becomes detached from the object, bit by bit, and attaches itself to the model” (Girard, 1987, p. 311).

A corollary of Girard's arguments relating to what he terms *mimetic desire* is that he characterizes human psychology as “interdividual” (Girard, 1987, p. 281ff.). That is, since our desires are dependent on the other, even to the extent of desiring to be like the other, our psychological disposition is not individually unique. And this understanding aligns with Mary Douglas's contention that the individual “needs fellow-consumers not only to create the social universe around him [*sic*] but to assure himself a tolerable place in it” such that “an individual's main objective in consumption is to help to create the social universe and to find in it a creditable place” (Douglas, 1976/2006, p. 242–243). And this also echoes Adam Smith's concerns for the “necessaries” of life such that “the poorest creditable person of either sex would be ashamed to appear in public without them” (Smith, 1776, p. 352, cited in Sen, 1984, p. 79).

This somewhat straightforward understanding of what lies behind desire—modeled on desires that we learn from others, and so as to find an unashamed and creditable place in the social world around us—does not, however, quite match with the rather more desperate and rampant nature of desire which some other commentators observe. Cushman (1990), for example, argued that the construction of the self, at least in the post-World War II American middle classes (though doubtless this could be extended to most contemporary consumer societies in the world), is of the “empty self”. In part because of “the loss of family, community, and tradition... it is a self that seeks the experience of being continually filled up by consuming goods, calories, experiences, politicians, romantic partners, and empathic therapists in an attempt to combat the growing alienation and fragmentation of its era” (Cushman, 1990, p. 600).^4^ As such, the self is “aggressively, sometimes desperately, acquisitive. It must consume in order to be soothed and integrated... or it will be in danger of fragmenting into feelings of worthlessness and confusion” (Cushman, 1990, p. 606).

We have so far articulated desire as being an individual or, perhaps more realistically, an “interdividual” affair. But Girard's insight that our desires are not original to ourselves can be extended from the individual level to considerations related to the economic order and the market, the state and conventional morality (MacIntyre, 2016). In relation to the first of these, MacIntyre argues that “the social order of capitalism... miseducates and wrongly directs desire” (MacIntyre, 2016, p. 108), such that agents “learn to want more and then more and then more and become consumed by their own desires” (MacIntyre, 2016, p. 109). This leads to the “paradox of capitalism” in that consumption is required to “serve the ends of expanding production”, and so “creates consumer societies in which its products can be successfully marketed only if the desires of consumers are directed toward whatever consumable objects the economy needs them to want” (MacIntyre, 2016, p. 109). Or, as Carrington et al. (2016, p. 22) put it: “consumption desires represent the false needs the system produces to chain us to endless processes of self-recreation and actualization through consumption”. And hence, desires for objects of consumption are shaped and elicited by “the seductive rhetoric of advertising and the deceptions of marketing” as a “necessary means for capitalist expansion” (MacIntyre, 2016, p. 109).^5^

Cushman noted that as advertising became ever more sophisticated, so credit (personal, business, and government) was needed to make “the new economy go” (Cushman, 1990, p. 603), and new industries—cosmetics, diet, electronic entertainment, preventive medical care and self-improvement (see Cushman, 1990, 604)—thereby came into being and expanded. As MacIntyre comments, “those who provide goods and services to such consumers shape their tastes so that they will desire and even take themselves to need whatever it is that that economy requires them to consume” (MacIntyre, 2016, p. 122, and note the subtle finessing of desire into need). He concludes that “the history of advertising and of public relations is too often a history of the misdirection of desire” (MacIntyre, 2016, p. 132), and this is reinforced by the advertising industry's concerns about consumer strategies for resisting advertising, and methods by which the industry might counter such strategies (Fransen et al., 2015).

But it is not just the economy and the market which direct consumers' desires. The “Morality” of modernity (“the moral system peculiar to and characteristic of early and late capitalist modernity”, MacIntyre, 2016, p. 115–116), also imposes “the requirement that we maximize well-being or happiness or some aspect of these” (MacIntyre, 2016, p. 65). And combining this concept of morality with the state and, as we have seen above, the market, MacIntyre concludes that the populace:

“... inhabit a social world structured to some large degree by the institutions of state, market, and Morality and find themselves in social relationships shaped directly and indirectly by these. *It is mostly taken for granted that what they want is what the dominant social institutions have influenced them to want*...” (MacIntyre, 2016, p. 166–167, emphasis added, although “desire” could just as well have been the term used, and would have been more consistent with MacIntyre's argument—see also below where the same inconsistency is observed).

However, while such institutions may well have a significant part to play in the education and determination of desires, this is not to say that these are the only influences. Significant among these other sources of the education of desire are families, schools and, potentially at least, workplaces (MacIntyre, 2016, p. 168–176). In these, desires for the internal goods of the practice (MacIntyre, 1981/2007, p. 187), and for common goods (MacIntyre, 2016, p. 170–175), noted above, are paramount. We will return to these below, but these desires, desires which, on MacIntyre's account, we have good reason to pursue, may, of course, conflict with other desires to which we are directed by the institutions of the market, state and Morality, and which the modern “interdividual” self, subject to mimetic desire, finds hard to resist.

Not only, then, do we perceive the “multifarious and heterogeneous sources of desire in the cultures of modernity and the consequent multifarious and heterogeneous desires” (MacIntyre, 2016, p. 132) which result for individuals, but we also see the need to order such desires. MacIntyre (2016, p. 133) poses the question in the following way:

“The cultures of modernity are cultures in which objects of desire and with them desires are multiplied. But along with the multiplication of desires comes a need to make choices. To which desires should I give priority? Which desires should I treat as realistic and which as vain wishes? How should I respond to conflicts of desire?”

MacIntyre's overtly NeoAristotelian answer to such questions is to turn to the concepts of goods, the good and human flourishing. In relation to whether a desire is a good desire, the initial question is, “Do I have sufficiently good reasons to want what I now want?” (MacIntyre, 2016, p. 4), which raises the further question as to whether acting in a particular way is likely to lead to some good which we are able to articulate. Thus, on this account, “we present ourselves to others, and they to us, as moved by desires for what we take to be goods. It is, insofar as the object of some particular desire really is a good, that it provides us with a good reason for acting so as to satisfy that desire” (MacIntyre, 2016, p. 11).

But this inevitably opens up the debate as to what “good” and goods are. And the answer which MacIntyre (2016, p. 29) offers is to do with human flourishing, interpreted in Aristotelian terms as to function well:

“... on the account of ‘good' and good that I am now outlining, if someone judges that it would be good for some particular individual or group to be, do, or have this or that, they are judging that for them to do, be or have this or that would contribute to human flourishing.”

It is important to note that MacIntyre insists that such a NeoAristotelian account allows for numerous ways in which flourishing may occur, and that therefore what it is to flourish in any particular set of circumstances “has to be discovered and often enough rediscovered” (MacIntyre, 2016, p. 30). A helpful distinction may be made here between “*the circumstances of a life* and *the living of a life*” (Annas, 2011, p. 92, emphasis in original) where the former is not under the agent's immediate control whereas the latter is. The issue then becomes a question of how to determine what human flourishing, in this or that particular set of circumstances of a life, consists of. And at this point the argument moves from the individual to the structural level: “For on any Aristotelian view we can only understand adequately what it is for human beings to flourish by developing an account of the structures of human activity and of how our uses of “good” and its cognates find application within and to those structures” (MacIntyre, 2016, p. 49).

And this move takes us to practices (MacIntyre's definition, as above) and the application of virtues, and the ways in which these enable us to learn what human flourishing consists in, all of which, as above, MacIntyre has already spelled out in *After Virtue* (1981/2007), and to which we will return below when considering the extended organizational framework. For now, we can simply note MacIntyre's answer to the question, how, then, do we “learn to distinguish what is good from what is taken to be good?” (MacIntyre, 2016, p. 49):

“We do so... in the context of a variety of practices, each with its own ends internal to it, generally first by learning how to contribute to the goods of the family and household in which we find ourselves and then learning how to do better or worse in the various activities of the school, the workplace, and the sports field... If all goes well, we develop in each area those habits, those dispositions, without which we cannot exercise the moral virtues. We also develop a habit of good practical judgment, the moral and intellectual virtue of prudence.^6^ Both types of habituation involve a transformation of desires and an increasingly sophisticated grasp of those standards, initially accepted on the authority of our teachers, by which they and hopefully we distinguish good from bad making and doing.” (MacIntyre, 2016, p. 49–50)

But this leaves open a further question. Given that, as MacIntyre emphasizes, the range of practices is wide (“arts, sciences, games, politics in the Aristotelian sense, the making and sustaining of family life, all fall under the concept,” MacIntyre, 1981/2007, p. 188), which practices should an individual agent give priority to in their life? And while the answer to this may vary over the course, and changed circumstances, of any individual life, it will be a common and consistent feature that the answer lies in shared deliberation over the goods that should be pursued, particularly given that in many cases “the goods in question are not individual but common goods, the goods of family, of political society, or workplace, of sports teams, orchestras, and theater companies” (MacIntyre, 2016, p. 51).

The argument so far, therefore, may be summarized in MacIntyre's words as follows:

“A single, if complex, theoretical conclusion [has] emerged. It is that agents do well only if and when they act to satisfy only those desires whose objects they have good reason to desire, that only agents who are sound and effective practical reasoners^7^ so act, [and] that such agents must be disposed to act as the virtues require...” (MacIntyre, 2016, p. 243)

## Virtuous organizations—The extended conceptual framework

The analysis to this point may well appear to have been mainly at the level of the individual and his or her desires, revealed in his or her patterns of consumption, and leading (or not) to his or her flourishing. But the structural nature of desire, and the social locations within which individual decisions to do with human flourishing are made, have also been evident. And, implicitly at least, there have been two core characterizations related to organizations in the material we have already covered, characterizations that enable the transition from the individual to the organizational level. First, in relation to consumer desire, there was a negative characterization associated with the goals of neo-liberal capitalism in general (the state, the market and present-day morality), and the credit, advertising and other industries in particular, which generate and generally misdirect desires. It is this characterization which leads not only to concerns over the creation of, and seductive impact on, the “empty selves” of individual lives, but also to the fact that the ratchet effect of desire is such that it may be leading us to the consuming of the very world on which we depend—climate change driven ultimately by consumer desire, as we shall see below.

Second, however, and countering this negative characterization, were references to workplaces (in addition to families and schools) as locations for MacIntyrean practices, in and through which desires could be (re)educated and potentially transformed in pursuit of the good. It is this positive characterization which offers the possibility of a significant extension to the current theory of virtuous organizations.

To see this in more detail, let us return initially to one of the key parts of the current theory, as noted above, which was the notion that the virtuous organization would necessarily have a good purpose as a core characteristic. In relation to extending the theory more needs to be said about this, in that recent work has been helpful in making this more concrete. Hsieh et al. (2018) distinguish between social purpose and what they term corporate (for which we could read ‘organization') purpose. Social purpose “concerns the contribution that a corporation makes to advancing societal goals, regardless of whether it directly pursues these goals or advances them as a side effect” (Hsieh et al., 2018, p. 52). Thus, a corporation provides employment and income (external goods) to its employees, and may thereby enable their personal development and even a sense of meaning in their lives, without this necessarily being an intentional aim—such that reducing levels of employment would be of no concern to the corporation.

There is a normative argument for requiring corporations to serve a social purpose. This argument is usually based on social contract theory (Donaldson and Dunfee, 1994; Hsieh, 2016) although a similar argument could be made from the requirement to pursue common goods noted above. Key here is the notion of reciprocity—that corporations benefit from the legal infrastructure, including that of limited liability, and the more general social infrastructure (transport, the prior education of employees, health services, and so on) which society provides. The very existence of corporations “is only possible through a societal license and continued support. In return, the operations of the corporation should have sufficient value for the society in which they operate” (Hsieh et al., 2018, p. 58). The same is true (generally with the exception of limited liability) for organizations in general. Of concern to this paper, as we shall see further below, and in practice to society in general, is the global ecological crisis, such that limiting the environmental impact of organizations is already, and will have to become even more so, a key social purpose: those that fail in this respect can in future expect their social license to operate to be revoked.

By contrast with social purpose, corporate purpose refers to “any non-financial social goals that the corporation directly pursues” (Hsieh et al., 2018, p. 52). This is not to say that corporate purpose is incompatible with pursuing profits (see Hsieh et al., 2018, p. 55), or more generally with financial sustainability, but implies that organizations might choose to pursue other purposes in addition to financial goals. It would, for example, be characteristic of the virtuous organization outlined above, that it would necessarily pursue goals in relation to its internal goods—the excellence of its products or services, the “perfection” of its practitioners—and the way in which both of these contribute to the realization of common goods. More generally, it has been argued that “focusing on purpose sets the challenge for all businesses in terms of promoting the common good” (Hollensbe et al., 2014, p. 1229).^8^

In relation to its practitioners, for example, the virtuous organization would see the provision of employment as one part of its corporate purpose, and further that their “perfection” through the provision of meaningful work (see Moore, 2017, p. 85–96, for example) would form another part. And similarly, it would be characteristic of the virtuous organization to pursue, as part of its “corporate” purpose, strategies which would minimize or eliminate its ecological impact, or better still be restorative of the environment as, again, we shall see in more detail below.

In summary, the way of conceptualizing the virtuous organization which has been outlined above places significant emphasis on the pursuit, ordering and balance in the achievement of three different kinds of goods—internal, external and common—and, through the establishment and achievement of corporate purpose, the broader pursuit of human flourishing including the enabling of a sustainable ecological environment. What, then, does this conceptualization imply in relation to human desire?

It was noted in the early sections of this paper that, while desire and the choice of which desires to pursue was, in a sense, an individual matter, these desires are shaped by the mimetic desire for objects that the ‘other' has, even to the extent of wishing to become like the other (Girard), and are also shaped by the dominant social institutions of the state, the market and present-day morality (MacIntyre). But amongst these social institutions are organizations themselves, which thereby also impose themselves upon us. And the morality of organizations in this sense, or at least of virtuous organizations as characterized above, requires that such organizations would be such as not to misdirect or mis-educate (the negative characterization above), but to *redirect and re-educate desire* (the positive characterization).

Before exploring what this means in practical detail, however, it is appropriate at this point to consider the application of this extended theory to the issue of climate change, and to compare it with solutions that have already been proposed in order to establish its unique contribution.

## Climate change: Challenges and potential solutions

Few would deny that we live in a fractured and uncertain world. In addition to, and to some extent framing, geopolitical machinations, the impact of climate change (IPCC, 2021, 2022a,b) and the associated loss of biodiversity and ecosystem services (IPBES, 2019) potentially threaten civilization as we know it. As the scientist Rovelli (2015, p. 76) has put it:

“We are perhaps the only species on earth to be conscious of the inevitability of our individual mortality. I fear that soon we shall also have to become the only species that will knowingly watch the coming of its own collective demise, or at least the demise of its civilization.”

The present age has been termed the Anthropocene, “a geological period characterized by a dominant human influence on the functioning of the ecosystem” (Ergene et al., 2021, p. 1320). While geologists continue to debate whether we have, indeed, moved into a new geological time period,^9^ it appears undeniable that “human activities are disrupting the functioning of the Earth as a complex, dynamic, ever-evolving totality comprised of myriad interlocking processes” (Hamilton, 2017, p. viii).^10^ These “Earth System” changes which are being experienced at the early stages of the Anthropocene epoch—fundamentally different from mankind's interaction with and impact on the environment to this point in history—indicate that “we are entering a new, unstable, and unpredictable geological era that will endure for thousands or tens of thousands of years” (Hamilton, 2017, p. 37).

As such, we need to understand the Anthropocene as “a very recent *rupture* in Earth history and so in human history” (Hamilton, 2017, p. 27, emphasis in original), and recognize that this represents “a scientific revolution akin to the Copernican Revolution” (Wright et al., 2018, p. 457), and thus a genuine paradigm shift with ontological meaning for the “joint human-Earth story” (Hamilton, 2017, p. 21) that we find we are writing, whether we like it or not.

Unsurprisingly, mainly as a result of climate change (though reinforced by the COVID-19 pandemic),^11^ the ecological, but also the ethical, origins and implications of the dominant neo-liberal capitalist system have been brought into question (see, for example, Collier, 2018). The urgent need to transition to a sustainable economy has become clear, but with it what has been referred to by the then Governor of the Bank of England as the ‘tragedy of the horizon': “once climate change becomes a defining issue for financial stability, it may already be too late” (Carney, 2015). In a later speech also from the Bank of England, the possibility that we might experience a “disorderly transition—with sudden, unanticipated and discontinuous effects, perhaps prompted by the greater occurrence of extreme weather events” was raised, together with an estimate of the finance required to effect the transition—in the region of $90tn by 2030, almost five times the GDP of the USA (Breeden, 2019).

Solutions have, of course, been proposed in response to the significant challenges summarized above. Among these there is a discourse that argues that climate change has already happened, and that we must therefore “first situate it as a discontinuity that renders unrecognizable everything that has come before” (Campbell et al., 2019, p. 737), such that “we are unable to subsume it into existing organizational thought and practice” (Campbell et al., 2019, p. 734). While recognizing this as a possibility, linked to the ontological paradigm shift and ‘tragedy of the horizon' noted above, this is clearly an extreme position which is not representative of the majority view that we do still have a chance, both organizationally and societally, of addressing the challenges that confront us. What, then, are the proposed solutions at the organizational level?

The quotation in the Introduction from Banerjee et al. (2021) referred to post-growth possibilities at the organization level. They note, however, the interchangeability of “post-growth” and “degrowth,” the latter being the more common term in the literature (see Latouche, 2009, for example). The degrowth movement defines sustainable degrowth as “a planned reduction of excess energy and resource use to bring the economy back into balance with the living world in a safe, just and equitable way” (Hickel, 2020, p. 29). It argues that “[even though] clean energy might help deal with emissions... it does nothing to reverse deforestation, over-fishing, soil depletion and mass extinction. A growth-obsessed economy powered by clean energy will still tip us into ecological disaster” (Hickel, 2020, p. 22). However, this is not just about *less*, including negative GDP growth, but also about *difference*: “different activities, different forms and uses of energy, different relations, different gender roles, different allocations of time between paid and non-paid work, different relations with the non-human world” (Kallis et al., 2015, p. 4). Similarly, while in aggregate there will be degrowth, there will be growth in some sectors (e.g., education, medical care and renewable energy) and shrinkage in others (e.g., so-called “dirty” industries, advertising and the financial sector)—hence, this is about *selective* degrowth.

However, as Robra et al. (2020) note, degrowth has received only marginal application at the organizational level and, we might add, also at the motivational aspects of the individual level. Banerjee et al., for example, while seeking to imagine post-growth organizations, present more of a summary of the problems of growth, and focus on alternative organizations and potentially SMEs, than the outlines of a solution at the organizational level (Banerjee et al., 2021, p. 345–347). An exception is Khmara and Kronenberg's (2018) proposal of seven criteria to assess whether a company is following a degrowth paradigm, and their preliminary analysis of Patagonia against these criteria. While there is some acknowledgment of what amounts to “having a good purpose,” and the need for broad educational campaigns on environmental justice and sustainability directed at customers and the local community (though surprisingly, not employees), there is a lack of connection with the underlying concerns for the analysis of desire and the motivating aspect of flourishing at the individual level, and the redirecting and re-educating responsibilities at the organization level, which are the focus of this paper.

Of more relevance to the organizational level is the ‘sufficiency' concept which both pre-dates and, in some cases (see Nesterova, 2020) aligns with the degrowth movement. Dyllick and Hockerts (2002) identified sufficiency as one of six criteria for corporate sustainability, although this was seen primarily as a corporate reaction to consumer and societal pressure. The concept was developed by Bocken and Short into sufficiency-driven business models. These are:

“about curbing consumption as part of the business model by moderating demand through education and consumer engagement, making products that last and avoiding built-in obsolescence; extending product lives to slow disposal and replacement, focusing on satisfying ‘needs' rather than promoting ‘wants' and fast-fashion, and reducing overall resource consumption through conscious changes in sales and marketing techniques, new revenue models, and innovative technology solutions.” (Bocken and Short, 2016, p. 46)

There are similarities here with the concept of the virtuous organization which has been developed above, and Bocken and Short provide a number of case studies to illustrate and explore the enablers and barriers to sufficiency-based business. As with degrowth, they note that there are likely to be individual winners and losers among organizations. Unlike Nesterova's (2020) exploration of a degrowth business framework, which has many similarities but where there is an acknowledgment that such a transition cannot be driven by business alone, Bocken and Short look to the business-case to drive sufficiency strategies. As such, this represents a market-led approach with no consideration of the significance of human desire in determining, and hence potentially mitigating, consumption.

Heikkurinen et al. (2019), like Nesterova (2020), look for action in the public sphere to promote “eco-sufficiency” strategies which focus on the quantity of economic activity, rather than just “eco-efficiency” strategies which focus on quality. Focusing on MNCs, they also argue for business to be proactive in encouraging consumers to consume less. Again, there are similarities with the virtuous organization approach developed above. But again the eco-sufficiency approach lacks the underlying analysis of individual desire and its fulfillment in consumption.

Sustainable consumption (and production if we align with No. 12 of the Sustainable Development Goals) provides a further element of this discourse (Welch and Southerton, 2019, for example). However, the focus is on responding to basic needs rather than desires—a distinction that was explored above—and on the policy approaches that are required to foster systemic change. While such policy approaches recognize the role that “key institutional actors” (Welch and Southerton, 2019, p. 40) might play, there is no consideration of organizations *per se* within this.

One final element of this discourse is around voluntary simplicity (Jackson, 2017, for example). Again, there is a combined focus at the individual level of “consciously minimizing wasteful and resource-intensive consumption” while also “reimagining ‘the good life'” (Alexander, 2015, p. 133). However, a critique of this approach is that it is “presented as a moral and individual matter. This is wrong: positional consumption is not a personal vice. It is a structural social phenomenon to which individuals conform to remain part of the mainstream” (Kallis, 2015, p. 138). Thus, solutions also need to be structural although Kallis takes these to be at the societal, not organizational, level. Jackson does address the organizational level, referring to “ecological enterprises” which “contribute positively to human flourishing; support community and provide decent livelihoods; and use as little as possible in the way of material and energy” (Jackson, 2015, p. 179). And while this takes us so far, in outline at least, it does not address the structural nature of human desire and how this might be handled at the organization level.

The conclusions that can be drawn from this review are that there is both a lack of any detailed consideration of the nature of human desire and how this reveals itself in patterns of consumption, and only a partial consideration of the organizational-level implications of the challenges which the Anthropocene poses. But, as we have noted, the extended theory of the virtuous organization deals precisely with these issues, and is therefore also a significant contribution to degrowth possibilities at the organization level (Banerjee et al., 2021).

What, then, are the practical implications of this extended theory of virtuous organizations, and what of its potential ubiquity? It is to these questions that we now turn.

## The extended theory in practice and countering an objection

There are two practical implications at the organizational level which we need to consider, and the second of these will lead us back to a consideration of social purpose and what may be required externally to support the internal elements of virtuous organizations. First, then, it is in the nature of such organizations that they involve for their employees (practitioners) an apprenticeship (see, for example, Moore, 2017, p. 62–63) into the practice which is at its core. And while such an apprenticeship will be partly to do with the techniques of the core practice, and the skills required to produce the associated goods or services, this will also be an apprenticeship into the nature of the practice itself, its history and the associated standards of excellence. This will be focused initially on the products or services themselves, and as such will be ‘on-the-job' training, but will then need to extend into a consideration of the virtues needed by practitioners in order to achieve these standards, and how these virtues might best be developed. This is, therefore, an apprenticeship first into the internal goods of the practice.

But it will then need to be an apprenticeship into the common goods of the community where practitioners deliberate “with others as to how in this particular set of circumstances here and now to act so as to achieve the common good of this particular enterprise” (MacIntyre, 2016, p. 174, cited above). This is, of course, as with internal goods, a significant educative task which will require skilled enablers, although these are skills and resources that the virtuous organization would normally find are already available internally and would be self-reinforcing over time.

There would, of course, also need to be an apprenticeship into the nature of external goods and how these arise for the particular organization and its core practice—its ‘business model.' And again, the virtuous organization would normally find that such skills and resources are already available internally. Putting these together, however, so that the inter-relationship between internal, common and external goods, their right ordering and their balanced pursuit over time, is understood would be a further requirement of the apprenticeship.

Put into other words, this would be an apprenticeship into what it is good to desire, an apprenticeship that would contribute to the development of the virtues, and thus toward practitioners becoming effective practical reasoners and, both individually and collectively, to the pursuit of human flourishing.

Furthermore, while apprenticeship is primarily focused on the organization's practitioners—its employees—there is an additional impact in relation to the education of the desires of its customers. It has been noted that the beneficiaries of the outputs of the practice—the customers who purchase the goods or services—may well be excellent judges of such output, and that they do, in some sense at least, determine the standards of excellence in the practice (Keat, 2000, p. 128–129). As such, they are also receiving an education into internal, common and external goods, an education that may also transform their desires. Virtuous organizations, as well as pursuing a good “corporate” purpose in relation to, amongst others, their own employees and their direct impact on the ecological environment, will, therefore, be in the business of redirecting and re-educating the desires of their employees and customers.

The second practical implication at the organization level follows in that, in order to effect this significant educative task, and further to actually put into practice the right ordering and balanced pursuit of internal, common and external goods by the organization itself, will require the development of power-balanced internal structures to off-set the natural tendency toward domination of the institutional element of the practice-institution combination. This will, in turn, require carefully designed systems of participation and self-governance to ensure that the views and desires of particular constituencies are not privileged over those of others, and will require decision-making systems and processes that enable rational critical dialogue to counter biases and allow questioning of the hitherto unquestioned (see MacIntyre, 1999b, p. 313; Moore, 2012b, p. 310).

It has already been noted that examples of such carefully designed systems of participation and self-governance examples may be found more readily in “coordinated” market economies such as Germany (Keat, 2008), and this takes us to the broader consideration of what may be required externally to support the internal elements of the virtuous organization. While it is clearly not impossible for any individual organization to put such educative and governance systems in place itself (see the example of Traidcraft given in Moore and Beadle, 2006), governments, in the provision of the broader governance arrangements and associated imposition of social purposes on organizations, clearly have a role here. It has been suggested, for example, that more democratic control in the economic sphere will not only have the effect of reducing inequality in levels of pay, but will also have a broader effect on consumerism and conspicuous consumption (Wilkinson and Pickett, 2019). Employee representatives on remuneration committees and boards, which are known to have beneficial effects in these directions (Wilkinson and Pickett, 2019, p. 248), together with community and consumer representatives on boards (Wilkinson and Pickett, 2019, p. 251), would in most cases require legislation to enforce.

These practical internal and external implications of virtuous organizations leads, however, directly to an obvious objection: surely, the approach being promoted here is utopian. It is *not* utopian in the sense that this could not be realized, indeed we already have examples that have at least moved in this direction (see Moore and Beadle, 2006; MacIntyre, 2016; Bernacchio, 2018), but is it utopian in the sense that it has little chance of becoming, as is proposed here, ubiquitous? There are, of course, reasons for thinking that lack of ubiquity will indeed be the case. In relation to climate change it has, for example, been noted how a process of organizational “hegemonization” allows “powerful corporations strategically [to] maintain a dominant ideology that downplays the need for radical climate change solutions” (Ferns and Amaeshi, 2021, p. 1006). And more generally, it has been noted how “grand challenges”, such as climate change, are “translated” by businesses “away from outcomes that challenge their profit-making abilities” (Wright and Nyberg, 2017, p. 1656). Indeed, even initiatives that might seem to be supportive of radical change, such as ethical consumption, have been shown, paradoxically, “to suppress a systemic critique of consumerist capitalism”, while maintaining an illusion that “actually closing this ethical gap would make a real difference to the destructiveness of the current system” (Carrington et al., 2016, p. 31).

Will the *status quo*, therefore, not continue, until we find ourselves at the very horizon we need to avoid, or can we find instead “a hopeful utopian discourse” (Schneider et al., 2010, p. 517), or what Latouche refers to as a concrete utopia which is both intellectually compelling and “takes as its starting point elements that already exist and changes that can be implemented” (Latouche, 2009, p. 42)? MacIntyre has, himself, commented similarly on a “utopianism of the present” in which, unlike utopias of the future that sacrifice the present to the future, “the range of present possibilities is always greater than the established order is able to allow for” (MacIntyre, 2011, p. 17). Is such a utopianism available?

A number of aspects of the above argument suggest that such a hopeful, concrete, present utopian discourse might indeed be available. Although the notion of paradigm change can be easily over-played, it does seem as though there is increasing recognition that a genuine paradigm shift, with ontological meaning for the “joint human-Earth story” (Hamilton, 2017, p. 21, cited above), is taking place.^12^ Indeed, the latest IPCC report noted demand-side interventions in relation to transport, sustainable healthy diets (see further below) and more generally: “Demand-side measures and new ways of end-use service provision can reduce global GHG [greenhouse gas] emissions in end use sectors by 40–70% by 2050 compared to baseline scenarios... Demand side mitigation response options are consistent with improving basic wellbeing for all” (IPCC, 2022b, p. 44).

Two aspects of the theory of desire covered above are also suggestive here. First, MacIntyre noted that, within an individual life, desires metamorphose; there is no ‘given' that present desires will remain the same. And in this connection, MacIntyre also noted that there is an essential future dimension to desire, so that actions now may determine the possibility of satisfying future desires in the medium- to long-term, and forgoing present desires may leave open future possibilities.

Second, the Girardian insights covered above suggest that, since desire is mimetic and our psychology “interdividual,” there is no reason why the ‘other' should not model for us desires that are different, and indeed more socially and ecologically sustainable, than those we currently have. Our models whose being we desire may be those who, for example, model voluntary simplicity to us. And there is no reason, on the above analysis, why it should not be virtuous organizations which so model for us, and so redirect and re-educate our desires toward a future desirable and sustainable state.

We also noted above that there tends to be a ‘ratchet' effect to consumerism, and that this seems always to be in one direction—ratcheting-up. But again, there is no reason, on the above analysis, why there should not be a *ratcheting-down* effect. A recent example is provided by the trend toward healthy diets from sustainable food systems (Willett et al., 2019), which promotes an equal concern for sufficient and healthy food for all in the world's population, while doing so in a manner that is consistent with the UN's Sustainable Development Goals and the 2015 Paris Climate Agreement. But the realization of such concerns will depend not only on action at individual, state and inter-governmental levels, but also on virtuous organizations which can contribute to the redirection and re-education of desires.

Finally, we should note the point made in the degrowth literature about *difference*. For some organizations in some sectors, where growth is anticipated (e.g., education, medical care and renewable energy), the redirection and re-education of desires ought to be relatively straightforward. The greater challenge, of course, is to those organizations and sectors where shrinkage is required. However, we already have extensive experience of industries which have declined or disappeared, of course—the coal industry in the UK being just one obvious example. And we have other more recent examples of successful transitions which reflect the necessity to achieve climate justice in the process (see Robinson, 2018). While acknowledging the level of ambition that is required, these examples and the trends identified above should enable organizations and sectors to transition to sustainable operations in all senses.

There might, then, on the analysis presented above, be a hopeful, concrete utopianism of the present, offering a greater range of present possibilities than the established order is able to allow for. And within that, virtuous organizations—all organizations—have a fundamental role to play.

## Conclusion, contributions and implications

The first aim of this paper was to build an extended theory of virtuous organizations, by integrating insights to do with human desire, consumption and human flourishing, drawn particularly from MacIntyre's latest book. In application, this extended theory is a response, in relation to the multiple challenges which society faces in the Anthropocene era, to the call for more theory building that articulates post-growth/degrowth possibilities at the organization level (Banerjee et al., 2021, p. 351).

The underlying analysis took us *via* a complex journey beginning with an analysis of desire at the individual level. From this understanding of desire we reviewed the fulfillment of those desires often through material consumption and, in the application to climate change, reached an understanding that it was the continual ratcheting-up of desire, coupled with the near-universal growth of consumer societies around the globe, which were at the heart of the ecological crisis that we face. We identified the need to understand how desires might be redirected and re-educated toward the good, toward human flourishing and the common good which also took into account ecological limits. And we saw how an extended version of the concept of virtuous organizations provided an answer at the organizational level which was significantly different from those proposed in the existing literature.

There are, of course, state-level aspects to the solution that has been formulated above. These might best be considered by inviting state-level (and potentially inter-governmental) action to define and enforce what we identified as the *social purpose* of organizations, possibly including more democratic control in the economic sphere, based on the reciprocal benefits that organizations already receive, and directed urgently and increasingly toward ecological sustainability.

From the perspective of organizations themselves, however, the answer has been formulated by laying out the parameters of virtuous organizations. Only by pursuing a *corporate* [organizational] *purpose* which meets these parameters can any organization individually, and organizations collectively, be considered to be responding appropriately to the challenges of the Anthropocene. The particular theoretical contribution of this paper, therefore, is to understand organizations as playing an important role in the redirection and re-education of desires, leading to the pursuit of goods that we have good reason to desire, and so to *the* good for individuals and communities, and ultimately to human flourishing within ecological limits for us all.

This contribution provides a philosophically- and practically-grounded basis on which existing approaches such as degrowth, eco-sufficiency, sustainable consumption, and voluntary simplicity can build. We also noted that critics will argue that this is a utopianism of the future, that while this might be possible for a small number of niche organizations, the neo-liberal economic system is such that organizations in general could not survive if they pursued the radical implications inherent here. But, as the challenges to the Earth System, and the implications of the Athropocene indicate, the time is such that we face a genuine paradigm shift to which organizations in general, and each organization in the particular, need to respond. Jackson (2017, p. 226–227) has put our situation like this:

“By the end of [this] century, our children and grandchildren will face a hostile climate, depleted resources, the destruction of habitats, the decimation of species, food scarcities, mass migrations and almost inevitably war.”

Virtuous organizations are part of the solution, if there is to be one, for our children and grandchildren.

But we do not have long.

## Data availability statement

The original contributions presented in the study are included in the article/supplementary material, further inquiries can be directed to the corresponding author.

## Author contributions

The author confirms being the sole contributor of this work and has approved it for publication.

## Conflict of interest

The author declares that the research was conducted in the absence of any commercial or financial relationships that could be construed as a potential conflict of interest.

## Publisher's note

All claims expressed in this article are solely those of the authors and do not necessarily represent those of their affiliated organizations, or those of the publisher, the editors and the reviewers. Any product that may be evaluated in this article, or claim that may be made by its manufacturer, is not guaranteed or endorsed by the publisher.

## Footnotes

^1^See also Sinnicks (2019) for an extended discussion of MacIntyre's concept of a practice.

^2^For other attempts to describe what is distinctive in the human condition, see Scruton (2017, p. 44).

^3^This is to distinguish a “person” from a “wanton” who is unconcerned “whether the [first-order] desires that move him [*sic*] to act are desires by which he wants to be moved to act” (Frankfurt, 1971, p. 12).

^4^For an analysis of the breakdown of the family, and prospects for the re-establishment of the “ethical family” see Collier (2018), Chapter 5.

^5^By no means do all share in the benefits of material consumption brought about by capitalism—see Collier (2018); it is a further paradox of capitalism that it depends on continual expansion but limits the ability of those at the “bottom” of society from being able to participate by limiting their remuneration.

^6^Elsewhere, MacIntyre refers to the achievement of such habits, dispositions and virtues as becoming an “independent practical reasoner” (see, MacIntyre, 1999a, p. 81ff. and further below where MacIntyre refers to “effective practical reasoners”, MacIntyre, 2016, p. 243).

^7^See footnote 6.

^8^This “From the Editors” article draws on an interview with Cardinal Vincent Nichols, Roman Catholic Archbishop of Westminster, UK; the common good is part of Catholic Social Teaching, and is linked to MacIntyre's usage of “common goods”.

^9^The International Commission on Stratigraphy (http://stratigraphy.org/) has yet to pronounce formally, but there appears to be general consensus among geologists that the changes to the “Earth System” are functionally and stratigraphically different to anything which has occurred in the (current) Holocene period. See Davidson (2019).

^1^That said, a critique of the concept of the Anthropocene is that it implicitly blames humanity *as a whole* for the problems we face, rather than acknowledging or challenging the capitalist system, with its associated “naturalized inequalities, alienation and violence inscribed in modernity's strategic relations of power and production” (Moore, 2015, p. 169), which is actually responsible. An alternative—Capitalocene—has been proposed which “does not stand for capitalism as an economic and social system” but “signifies capitalism as a way of organizing nature—as a multispecies, situated, capitalist world ecology” (Moore, 2016, p. 6).

^11^Working on earlier drafts of this article during the COVID-19 coronavirus pandemic has helped to underline the severity of the situation we face. The economic effects of the virus lowered the impact on the ecological environment through a reduction in carbon emissions. The International Energy Agency (IEA) estimates, however, that the effect of the virus on energy demand in 2020 compared with 2019 was a contraction of just 4%, with global CO_2_ emissions declining by 5.8% (IEA, 2021—the greater decline in CO_2_ emissions over energy demand was due to an increase in the use of renewable over fossil fuels). By December 2020 global CO_2_ emissions were 2% *higher* than in December 2019, and further increased by 6% in 2021 (IEA, 2022). Comparing this with the IPCC (2018) projection that CO_2_ emissions will need to be reduced to net zero globally by 2050, with substantial reductions in emissions from 2020 onwards if global warming is to be limited to 1.5°C, and the size of the task becomes evident.

^12^While it is somewhat early for considered reviews in the academic literature of the effects of the COVID-19 pandemic, there have already been calls on policy-makers to take the opportunity “to reduce over the longer term the prevalence of lifestyles premised on large volumes of energy and material throughput” [Cohen, 2020, p. 2, and see also IEA, 2021 and the UK Committee on Climate Change report “Progress in reducing emissions. 2021 Report to Parliament” available at 2021 Progress Report to Parliament—Climate Change Committee (theccc.org.uk), accessed 27 September 2022]. In relation to consumption, the possibility of “post-material frugality” has been proposed as a lifestyle variation arising out of the pandemic (Echegaray, 2021, p. 571). But it now seems unlikely that the pandemic will serve as a trigger-point in the shift of paradigm that is required.
